# Hydroxysafflor Yellow A Suppresses MRC-5 Cell Activation Induced by TGF-β1 by Blocking TGF-β1 Binding to TβRII

**DOI:** 10.3389/fphar.2017.00264

**Published:** 2017-05-11

**Authors:** Ruiyan Pan, Yadan Zhang, Meng Zheng, Baoxia Zang, Ming Jin

**Affiliations:** Department of Pharmacology, Beijing Anzhen Hospital, Capital Medical University, Beijing Institute of Heart Lung and Blood Vessel DiseasesBeijing, China

**Keywords:** hydroxysafflor yellow A, TGF-β1, TβRII, Smad, fibroblast

## Abstract

Hydroxysafflor yellow A (HSYA) is an active ingredient of *Carthamus tinctorius* L.. This study aimed to evaluate the effects of HSYA on transforming growth factor-β1 (TGF-β1)-induced changes in proliferation, migration, differentiation, and extracellular matrix accumulation and degradation in human fetal lung fibroblasts (MRC-5), to explore the mechanisms whereby HSYA may alleviate pulmonary fibrosis. MRC-5 cells were incubated with various doses of HSYA and/or the TGF-β receptor type I kinase inhibitor SB431542 and then stimulated with TGF-β1. Cell proliferation was measured by 3-(4,5-dimethylthiazol-2-yl)-5-(3-carboxymethoxyphenyl)-2-(4-sulfo-phenyl)-2H-tetrazolium inner salt assay. Cell migration was detected by wound-healing assay. Protein levels of α-smooth muscle actin (α-SMA), collagen I α 1 (COL1A1), and fibronectin (FN) were measured by immunofluorescence. Protein levels of matrix metalloproteinase-2, tissue inhibitor of matrix metalloproteinase-1, tissue inhibitor of matrix metalloproteinase-2, TGF-β type II receptor (TβRII), and TGF-β type I receptor were detected by western blotting. TβRII knockdown with siRNA interfered with the inhibitory effect of HSYA on α-SMA, COL1A1, and FN expression, and TGF-β1-induced Sma and Mad protein (Smad), and extracellular signal-regulated kinase/mitogen-activated protein kinase signaling pathway activation. The antagonistic effect of HSYA on the binding of fluorescein isothiocyanate-TGF-β1 to MRC-5 cell cytoplasmic receptors was measured by flow cytometry. HSYA significantly suppressed TGF-β1-induced cell proliferation and migration. HSYA could antagonize the binding of FITC-TGF-β1 to MRC-5 cell cytoplasmic receptors. Also HSYA inhibited TGF-β1-activated cell expression of α-SMA, COL1A1, and FN and phosphorylation level of Smad2, Smad3, and ERK by targeting TβRII in MRC-5 cells. These findings suggest that TβRII might be the target responsible for the inhibitory effects of HSYA on TGF-β1-induced pathological changes in pulmonary fibrosis.

## Introduction

Idiopathic pulmonary fibrosis (IPF) is defined as chronic, unrelenting, and progressive fibrosing interstitial pneumonia with unknown cause (Raghu et al., 2011; Puglisi et al., 2016). The number of patients suffering IPF has doubled within the past decade, and the overall prognosis is dismal, with a median survival of 3–5 years following diagnosis (Verma and Slutsky, 2007). Increasing evidence indicates that IPF is caused by the abnormal behavior of alveolar epithelial cells, which provoke fibroblast proliferation, migration, and differentiation. Activated fibroblasts secrete increased extracellular matrix (ECM) molecules, resulting in subsequent destruction of the lung architecture (King et al., 2011).

Transforming growth factor-β (TGF-β) is a pleiotropic factor that regulates cell differentiation and growth, tissue homeostasis and repair, and immune and inflammatory responses (Biernacka et al., 2011). It is considered to be a crucial molecule involved in the activation of the fibrosis (Leask and Abraham, 2004). Active levels of TGF-β1 were reportedly increased in the lungs of IPF patients and in a bleomycin (BLM)-induced pulmonary fibrosis model (Coker et al., 1997, 2001; Shimbori et al., 2013; Wolters et al., 2014). Inhibitors of the TGF-β1 signaling pathway have thus emerged as potential therapies for IPF.

*Carthamus tinctorius* L. has been used extensively as a traditional herbal medicine in China to treat various conditions, including gynecological, cardiovascular, and cerebrovascular diseases, as well as blood stasis and osteoporosis (Li et al., 2009; Han et al., 2016; Wang et al., 2016; Zhang et al., 2016). Over 100 compounds have been isolated from *C. tinctorius* L. and identified (Zhang et al., 2016), including hydroxysafflor yellow A (HSYA) as one of main active constituents. Injection of safflor yellow (the main active ingredient of which is HSYA) achieved remarkable clinical effects in patients with cardiovascular and cerebrovascular diseases in China (Zhang et al., 2005; Yang et al., 2009; Li et al., 2015). HSYA has also demonstrated other pharmacological activities, including antioxidant, anti-inflammatory, and antitumor activities (Yang F.F. et al., 2015; Chen et al., 2016; Cheng et al., 2016; Han et al., 2016; Xu et al., 2016), and has also shown potential as a therapeutic candidate for fibrosis. HSYA attenuated carbon tetrachloride-induced hepatic fibrosis by inhibiting activation of hepatic stellate cells and attenuating TGF-β signaling (Zhang et al., 2012). HSYA also ameliorated renal fibrosis by suppressing TGF-β1-induced epithelial-to-mesenchymal transition (Hu et al., 2016). We previously reported that HSYA could attenuate BLM-induced pulmonary fibrosis (Jin et al., 2016) and inhibit TGF-β1-induced activation of human fetal lung fibroblasts (Pan et al., 2016), but the precise mechanisms and target of HSYA’s anti-fibrosis activity remain unclear.

In this study, we investigated the effects of HSYA on TGF-β1-induced changes in cell proliferation, migration, differentiation, and ECM synthesis and degradation in human fetal lung fibroblasts, and explored the mechanisms and target of HSYA.

## Materials and Methods

### HSYA Preparation

*Carthamus tinctorius* L. was grown in Tacheng, Xinjiang Uygur Autonomous Region, China, and the flowers (safflower) were provided by Huahui Kaide Pharmaceutical Co., Ltd (Shanxi, China) and identified by Professor Jiashi Li (Beijing University of Traditional Chinese Medicine). HSYA was isolated and purified from the aqueous extract of *C. tinctorius* L. by macroporous resin-gel column chromatography, as described previously (Zang et al., 2008). The molecular weight of HSYA is 612 and the molecular structure has been previously described by Feng et al. (2013). HSYA was diluted with aseptic 0.9% NaCl for experimental use. HSYA analysis was performed using a high-performance liquid chromatography system (Wang Y. et al., 2014). The purity of the HSYA was previously determined by the area normalization method to be 95.2% (Pan et al., 2016).

### Experimental Reagents

Recombinant human TGF-β1 (PeproTech Company, Rocky Hill, NJ, USA) was dissolved in phosphate-buffered saline (PBS) containing 5% trehalose. The TGF-β receptor type I kinase inhibitor, SB431542 (Sigma–Aldrich, St. Louis, MO, USA), was diluted with dimethylsulfoxide (DMSO) to a final concentration of DMSO of 0.1% in the reaction system. Fluorescein isothiocyanate (FITC)-labeled TGF-β1 was synthesized and purified by dialysis against PBS pH 7.4 by Beijing Fanbo Biochemicals Co. Ltd, as described previously (Xia et al., 1996). TGF-β type II receptor (TβRII) siRNA, non-targeting control siRNA, siRNA transfection reagent, α-smooth muscle actin (α-SMA), fibronectin (FN), TβRII, and TGF-β type I receptor (TβRI) antibodies were from Santa Cruz Biotechnology (Santa Cruz, CA, USA). Collagen I α 1 (COL1A1) antibody was from Novus Biological (Littleton, CO, USA). Human matrix metalloproteinase-2 (MMP-2), tissue inhibitor of matrix metalloproteinase-1 (TIMP-1), and TIMP-2 antibodies were from R&D Systems (Minneapolis, MN, USA). Phospho-Smad2 (Ser465/467), phospho-Smad3 (Ser423/425), Smad2/3, phospho-p44/42 mitogen-activated protein kinase (MAPK) (extracellular signal-regulated kinase 1/2; ERK1/2) (Thr202/Tyr204), and p44/42 MAPK (ERK1/2) primary antibodies were from Cell Signaling Technology (Danvers, MA, USA). The 3-(4,5-dimethylthiazol-2-yl)-5-(3-carboxymethoxyphenyl)-2-(4-sulfo-phenyl)-2H-tetrazolium inner salt (MTS) assay kit was from Promega (Madison, WI, USA), the SYBR^®^ Premix Ex Taq^TM^ (Perfect Real Time) kit was from Agilent Technologies (Santa Clara, CA, USA), and glyceraldehyde phosphate 3-dehydrogenase (GAPDH) antibody, RIPA lysis buffer, phenylmethanesulfonyl fluoride, and the bicinchoninic acid assay kit were from Beyotime Institute of Biotechnology (Jiangsu, China).

### Cell Culture

MRC-5 human fetal lung fibroblasts were obtained from the Institute of Basic Medicine, Chinese Academy of Medical Sciences, and cultured in minimum essential medium (Thermo Scientific, Walton, MA, USA) supplemented with 10% fetal bovine serum (Thermo Scientific), 1% non-essential amino acids (Keygen, China), 100 g/mL streptomycin, and 100 U/mL penicillin (Thermo Scientific) at 37°C, in a 5% CO_2_ incubator.

### Experimental Groups

In the first part of the study, we assessed the effects of HSYA on TGF-β1-induced cell proliferation, migration, and expression levels of MMP-2, TIMP-1, TIMP-2, TβRII, and TβRI. Cells were divided into eight groups as follows: normal control; HSYA blank control (45 μmol/L); TGF-β1; TGF-β1+HSYA (5, 15, and 45 μmol/L); TGF-β1+SB431542; and TGF-β1+SB431542+HSYA (45 μmol/L). Cells in the TGF-β1+SB431542+HSYA (45 μmol/L) group were treated with HSYA, SB431542, and TGF-β1. In the TGF-β1+SB431542 group, an equivalent volume of 0.9% NaCl was added instead of HSYA; an equivalent volume of DMSO was added instead of SB431542 in the TGF-β1+HSYA (5, 15, and 45 μmol/L) groups; and equivalent volumes of 0.9% NaCl and DMSO were added instead of HSYA and SB431542, respectively, in the TGF-β1 group. In the HSYA blank control, equivalent volumes of PBS containing 5% trehalose and DMSO were added instead of TGF-β1 and SB431542, and the normal control included equivalent volumes of 0.9% NaCl, PBS containing 5% trehalose, and DMSO instead of HSYA, TGF-β1, and SB431542, respectively.

The second part of the experiment was performed to confirm the inhibitory effects of HSYA on TGF-β1-induced α-SMA, COL1A1, and FN expression. For this, six treatment groups were established as follows: normal control; HSYA blank control (45 μmol/L); TGF-β1; TGF-β1+HSYA (45 μmol/L); and TGF-β1+SB431542. Cell treatments were as above.

The third part of the experiment was conducted to detect the effects of HSYA on expression levels of α-SMA, COL1A1, and FN, and on phosphorylation of Smad2, Smad3, and ERK following TβRII knockdown. Cells were divided into nine groups as follows: normal-untransfected; normal-non-targeting control siRNA; normal-TβRII siRNA; TGF-β1-untransfected; TGF-β1-non-targeting control siRNA; TGF-β1-TβRII siRNA; (TGF-β1+HSYA)-untransfected; (TGF-β1+HSYA)-non-targeting control siRNA; and (TGF-β1+HSYA)-TβRII siRNA. The normal-untransfected, TGF-β1-untransfected, and (TGF-β1+HSYA)-untransfected groups were treated the same as the normal, TGF-β1, and TGF-β1+HSYA (45 μmol/L) groups in the first part of the experiment, respectively. The normal-TβRII siRNA, TGF-β1-TβRII siRNA, and (TGF-β1+HSYA)-TβRII siRNA groups first underwent TβRII knockdown, and were then treated like the normal, TGF-β1, and TGF-β1+HSYA (45 μmol/L) groups in the first part of the experiment, respectively. The normal-non-targeting control siRNA, TGF-β1-non-targeting control siRNA, and (TGF-β1+HSYA)-non-targeting control siRNA groups received non-targeting control siRNA instead of TβRII siRNA.

### MTS Assay

Cells were seeded in 96-well plates at a density of 3 × 10^3^ cells/well and cultured for 24 h. After 12 h of serum starvation, the cells were pretreated with various doses of HSYA or 2 μmol/L SB431542 before stimulation with 1 ng/mL TGF-β1. A blank well with the same volume of medium but without cells was treated simultaneously, as a control well. After incubation for 24, 48, or 72 h, 20 μL CellTiter 96 Aqueous One Solution Reagent was added for 1 h at 37°C, and the optical density (OD) at 490 nm was then measured using a microplate reader (BioTek, Winooski, VT, USA).

### Wound-Healing Assay

To establish a grid for orientation, in each well of a six-well plate, we first drew five lines parallel to the long edges of the plate. Then cells were cultured in these labeled six-well plates for 24 h to reach 80–90% confluency. After serum starving for 12 h, the cell layer in the middle of the well was scratched perpendicularly to the five lines using a pipette tip and a wounded region formed. The wounded region and five transverse lines crossed each other to form a grid which was used to follow cell migration in the same field over time. The size of the regions within each grid was approximately the same as the microscopic field of view at 50× magnification. Subsequently, cells were washed twice with PBS and then treated with various doses of HSYA or 2 μmol/L SB431542 for 30 min before stimulation with TGF-β1. Images of the wounded region from the same sector of the grid were acquired at 0, 24, and 48 h following treatment using an inverted microscope (Leica, Germany). The area and height of the rectangular wounded region were calculated using Image J software. The area divided by height gave the width. Cell migration rates were calculated as follows:

$$\begin{array}{c} \mathrm{migration}{\mathrm{rate}}_{24\;h\;\mathrm{or}\;48\;h}(\%)\;\;=\;[({\mathrm{width}}_{0\;h}\;-\;{\mathrm{width}}_{24\;h\;\mathrm{or}\;48\;h})/{\mathrm{width}}_{0\;h}]\;\times \;100\% \end{array}$$

### Immunofluorescence

MRC-5 cells were grown on sterile glass slides for 24 h in 6-well plates to reach 80–90% confluency and then serum starved for 12 h. At that time, cells were treated with 45 μmol/L HSYA or 2 μmol/L SB431542 for 30 min before stimulation with TGF-β1 for 72 h. After washing three times with cold PBS, the cells were immobilized with 4% paraformaldehyde (Thermo Scientific) for 15 min and permeabilized by 0.1% Triton X-100 in PBS for 10 min. The cells were then incubated for 30 min in blocking buffer (3% bovine serum albumin/PBS) at 37°C and stained with primary antibodies recognizing α-SMA, COL1A1, and FN diluted in blocking buffer at 4°C overnight, followed by incubation with FITC-labeled goat anti-rabbit or anti-mouse antibody (Beyotime Institute of Biotechnology) at 37°C for 2 h. Cell nuclei were stained with DAPI (Beijing, China) at room temperature for 5 min. Immunofluorescent images were captured using a fluorescence microscope (Nikon 80i, Japan).

### siRNA Silencing

Cells were plated in six-well plates for 24 h to reach 80–90% confluency and then incubated with serum-free medium for 12 h. Five microliters of siRNA (TβRII or non-targeting control) solution were mixed with 100 μL siRNA transfection medium to create reagent A, and 5 μL transfection reagent was mixed with 100 μL siRNA transfection medium to create reagent B. Reagents A and B were then mixed to give reagent C and kept at room temperature for 30 min. After washing with transfection medium, 200 μL reagent C and 800 μL transfection medium were added to the cells and incubated at 37°C for 6 h. One milliliter of medium containing 20% fetal bovine serum was then added to each well and the cells were incubated for 24 h, followed by the addition of 45 μmol/L HSYA, incubation for 30 min, and stimulation with TGF-β1.

### Real-Time PCR Analysis

Total RNA was isolated from MRC-5 cells using TRIzol reagent (Invitrogen, Carlsbad, CA, USA), according to the manufacturer’s instructions. The RNA concentration and quality were measured using a NanoDrop 2000 (Thermo Scientific, Wilmington, DE, USA). Two micrograms of RNA were reverse transcribed to cDNA using a cDNA reverse transcriptase kit (Promega, Madison, WI, USA). Real-time PCR was performed using a SYBR^®^ Premix Ex Taq^TM^ Kit with a Bio-Rad iCycler iQ5 Detection System (Bio-Rad, Hercules, CA, USA). The PCR amplification conditions were as described previously (Pan et al., 2016). The primer sequences and lengths (in bp) were as follows (5′–3′): MMP-2: TGAGCTATGGACCTTGGGAGAA (forward) and CCATCGGCGTTCCCATAC (reverse), 60 bp; TIMP-1: CTGTTGTTGCTGTGGCTGATAG (forward) and AAGGTGGTCTGGTTGACTTCTG (reverse), 137 bp; TIMP-2: GGGAAGGATTTTGGAGGTAGG (forward) and GGAGGCTGAGAAAGAAGTGAGTG (reverse), 58 bp. The threshold cycle (Ct) was automatically calculated and displayed in the PCR instrument after each cycle. Relative quantities of mRNA were calculated using the 2^-ΔΔCt^ method, normalized to the GAPDH gene.

### Western Blot Analysis

MRC-5 cells were lysed with RIPA lysis buffer mixed with 1 μM phenylmethanesulfonyl fluoride and 1 μM phosphatase inhibitor cocktail 3 (Sigma–Aldrich). Cell protein concentrations were measured using a bicinchoninic acid assay kit. Protein samples were isolated by sodium dodecyl sulfate-polyacrylamide gel electrophoresis and then transferred to nitrocellulose membranes. After blocking with 5% non-fat milk, the membranes were incubated at 4°C overnight with antibodies recognizing the following antigens: α-SMA, COL1A1, FN, MMP-2, TIMP-1, TIMP-2, TβRII, TβRI, p-Smad2, p-Smad3, p-ERK, Smad2/3, ERK, and GAPDH (1:500–1000 dilution). After three washes with Tris-buffered saline-Tween, the membranes were incubated with IR Dye^®^-conjugated goat anti-mouse, goat anti-rabbit, or donkey anti-goat antibodies (1:5000 dilution, LI-COR Biosciences, Lincoln, NE, USA) for 1 h. The band intensities were scanned and quantified using an Odyssey infrared imaging system (Gene Company, Beijing, China).

### Specific Receptor Binding Assay

MRC-5 cells at a density of 1 × 10^6^ cells/well were divided into six groups: normal; FITC-TGF-β1 (20 ng/μL); FITC-TGF-β1 (20 ng/μL)+HSYA (5, 15, or 45 μmol/L); and FITC-TGF-β1 (20 ng/μL)+TGF-β1 (2 μg/μL). Cells in the FITC-TGF-β1+HSYA group were treated with HSYA and FITC-TGF-β1. In the FITC-TGF-β1+TGF-β1 group, HSYA was replaced with TGF-β1 solution (final concentration 2 μg/μL); in the FITC-TGF-β1 group, HSYA was replaced with 0.9% NaCl; and in the normal group, HSYA and FITC-TGF-β1 were replaced with 0.9% NaCl and PBS, respectively. After the reagents were added, the cell suspensions were incubated at 37°C for 40 min with shaking at 300 rpm using an orbital shaker (Eppendorf, Germany). The cells were subsequently cooled at 4°C for 10 min, centrifuged at 1000 rpm for 5 min, and washed twice with PBS. The precipitate was then suspended in 300 μL PBS and detected by flow cytometry (Beckman Coulter, Brea, CA, USA). The mean fluorescence intensity (gate %) showed the specific binding intensity of FITC-TGF-β1 to MRC-5 cell membrane receptors.

### Statistical Analysis

Data analysis was performed using SPSS 13.0 (SPSS Inc., Chicago, IL, USA) and GraphPad Prism 5.0 (GraphPad Software, Inc., USA) software. Multiple comparisons were made by one-way analysis of variance (ANOVA) with Student–Newman–Keuls *post hoc* tests. Data were presented as mean ± SD. A *p*-value < 0.05 was defined as statistically significant.

## Results

### HSYA Inhibited TGF-β1-Induced MRC-5 Cell Proliferation and Migration

Previously, we showed that MRC-5 cells treated with HSYA for 72 h could inhibit TGF-β1-induced cell proliferation in a dose-dependent manner (Pan et al., 2016). To determine how early this effect appeared, we monitored the effect of HSYA on TGF-β1-induced cell proliferation after 24, 48, or 72 h. As depicted in **Supplementary Figure S1**, the OD measure of proliferation of MRC-5 cells was lower in the TGF-β1+HSYA treated group as compared to the group treated with TGF-β1 alone after 48 or 72 h. This effect was dose dependent. However, after treatment with 5, 15, and 45 μmol/L HSYA for 24 h, we could not find any significant change in the OD value. Further, the OD values for the TGF-β1+SB431542 treated cells were comparable to those of the TGF-β1+SB431542+HSYA treated group, suggesting that the effect of HSYA was dependent on the TGF-β receptor type I kinase pathway.

**FIGURE 1:**
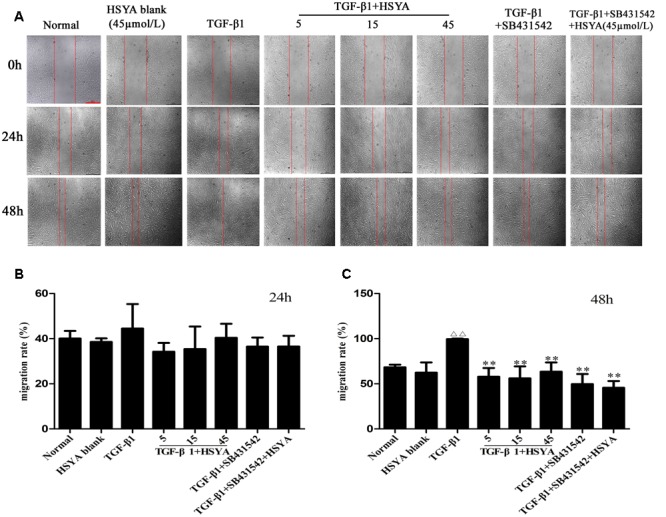
**Effects of hydroxysafflor yellow A (HSYA) on TGF-β1-induced MRC-5 cell migration.** Migration of MRC-5 cells treated with HSYA (5, 15, and 45 μmol/L) and/or 2 μmol/L SB431542 and then stimulated with 10 ng/mL TGF-β1 at 24 and 48 h was measured by wound-healing assay. **(A)** Images were captured by inversion fluorescence microscopy (magnification: 50×, scale bar = 500 μm). Migration rates of cells at 24 **(B)** and 48 h **(C)** of wound-healing assay. Data presented as mean ± SD, *n* = 4 per group. ^ΔΔ^*p* < 0.01 vs. normal group, ^∗∗^*p* < 0.01 vs. TGF-β1 group.

Because HSYA affected the TGF-β1 induced proliferation of MRC-5 cells, we hypothesized that it might also influence other TGF-β1-mediated processes like cell migration. To that end, we assessed the effects of HSYA on TGF-β1-induced cell migration by wound-healing assay (**Figure 1A**). Consistent with our cell proliferation data, there was no significant difference in cell migration between the TGF-β1+HSYA and TGF-β1 groups at 24 h (**Figure 1B**). However, when the wound was examined after 48 h, we found that the TGF-β1-induced migration of MRC-5 cells was significantly inhibited when treated with HSYA as compared to those cells treated with TGF-β1 alone (**Figure 1C**). Further, cell migration was similar in the TGF-β1+SB431542 and TGF-β1+SB431542+HSYA groups, suggesting that the effect of HSYA was dependent on the TGF-β receptor type I kinase pathway. Taken together, our data show that treatment of MRC-5 cells with HSYA for 48 h can attenuate both the TGF-β1-induced proliferation and migration response, although this effect is not apparent at 24 h.

### Effect of HSYA on MRC-5 Cell Differentiation to Myofibroblasts and Resulting ECM Accumulation and Degradation

The pathology of IPF is thought to arise, in part, from the conversion of fibroblasts into myofibroblasts, which express α-SMA and secrete ECM proteins. Increased deposition of ECM and reduced matrix degradation are the underlying causes of IPF. We have shown that HSYA decreased the elevated mRNA and protein expression of α-SMA, COL1A1, and FN induced by TGF-β1 (Pan et al., 2016). Building upon this observation, we performed immunofluorescent staining of MRC-5 cells treated with HSYA, with TGF-β1, with TGF-β1+HSYA, or with TGF-β1+SB431542 and compared the expression of α-SMA, COL1A1, and FN to untreated cells. As seen in **Supplementary Figure S2**, TGF-β1 treatment increased the intracellular incorporation of α-SMA-positive filaments into MRC-5 cells and strongly upregulated the expression of COL1A1 and FN staining along the cell axis as compared to untreated cells. This change was abrogated with either TGF-β1 inhibition (via SB431542 treatment) or with HSYA treatment, indicating that HSYA can modulate the TGF-β1-induced differentiation of MRC-5 cells into myofibroblast-like cells as well as the resulting increase in synthesis of ECM proteins.

**FIGURE 2:**
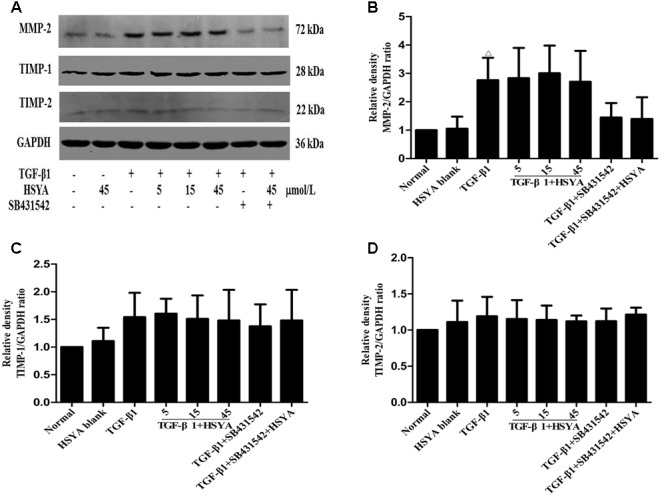
**Effects of HSYA on MMP-2, TIMP-1, and TIMP-2 expression in TGF-β1-activated MRC-5 cells.** MRC-5 cells were pretreated with HSYA and/or 2 μmol/L SB431542 and then cultured with 10 ng/mL TGF-β1 for 72 h. Protein expression levels were measured by western blotting **(A)**. Densitometric analyses of MMP-2 **(B)**, TIMP-1 **(C)**, and TIMP-2 **(D)**. Data are presented as mean ± SD, *n* = 4 per group. ^Δ^*p* < 0.05 vs. normal group.

To determine whether HSYA could influence the degradation of the ECM, we measured the protein levels of MMP-2, TIMP-1, and TIMP-2 by western blotting. As shown in **Figure 2**, MMP-2 protein levels increased when MRC-5 cells were stimulated with TGF-β1 for 72 h compared with the normal group. However, there was no significant increase in TIMP-1 or TIMP-2 expression compared with the normal group. HSYA treatment had no effect on MMP-2, TIMP-1, or TIMP-2 protein expression levels. In total, our data indicate that while HSYA can attenuate the TGF-β1-induced transdifferentiation of MRC-5 fibroblasts into myofibroblast-like cells and associated synthesis of ECM proteins, it has no effect on the degradation of the ECM.

### Effects of HSYA on TGF-β1-Induced TβRII and TβRI Expression in MRC-5 Cells

**FIGURE 3:**
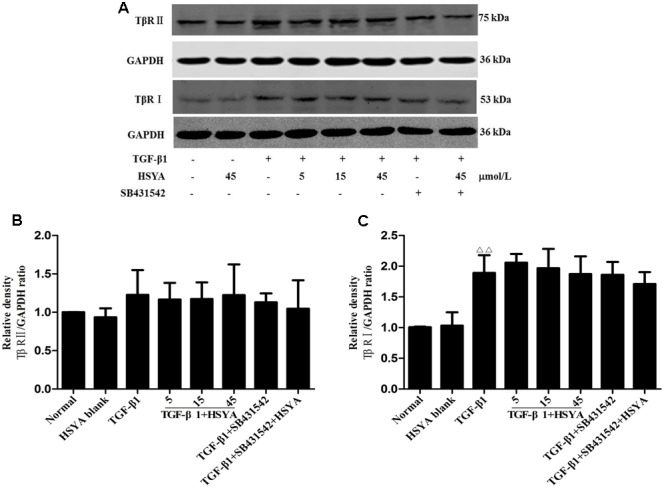
**Effects of HSYA on TβRII and TβRI expression in TGF-β1-treated MRC-5 cells.** Cells were pretreated with 5, 15, or 45 μmol/L HSYA and/or 2 μmol/L SB431542 then stimulated with 10 ng/mL TGF-β1 for 24 h. Protein levels of TβRII and TβRI were measured by western blotting **(A)**. Densitometric analyses of TβRII **(B)** and TβRI **(C)**. Data are presented as mean ± SD, *n* = 4 per group. ^ΔΔ^*p* < 0.01 vs. normal group.

Protein levels of TβRI increased after activation by TGF-β1, while TβRII expression levels were not obviously increased. HSYA treatment had no effect on the expression of TβRII or TβRI (**Figure 3**).

### Effects of HSYA on α-SMA, COL1A1, and FN Expression in TGF-β1-Treated MRC-5 Cells after TβRII Knockdown

**FIGURE 4:**
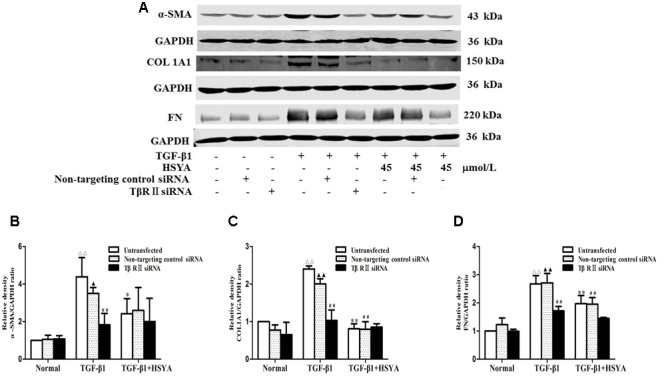
**Effects of HSYA on α-SMA, COL1A1, and FN protein expression in TGF-β1-treated MRC-5 cells with TβRII knockdown.** MRC-5 cells were pretreated with TβRII-non-targeting control siRNA or TβRII siRNA for 24 h followed by 45 μmol/L HSYA for 30 min before stimulation with 10 ng/mL TGF-β1 for 48 h. Protein expression levels of α-SMA, COL1A1, and FN were measured by western blotting **(A)**. Densitometric analyses of α-SMA **(B)**, COL1A1 **(C)**, and FN **(D)**. Data are presented as mean ± SD, *n* = 3 per group. ^ΔΔ^*p* < 0.01 vs. normal-untransfected group, ^▴^*p* < 0.05, ^▴▴^*p* < 0.01 vs. normal-non-targeting control siRNA group, ^∗^*p* < 0.05, ^∗∗^*p* < 0.01 vs. TGF-β1-untransfected group. ^##^*p* < 0.01 vs. TGF-β1-non-targeting control siRNA group.

We performed knockdown of TβRII with siRNA to evaluate if HSYA suppressed TGF-β1-induced cell transdifferentiation and ECM accumulation by targeting TβRII on the cell membrane. Protein levels of α-SMA, COL1A1, and FN were significantly increased in the TGF-β1-untransfected group compared with the normal-untransfected group, and these increases were attenuated in the (TGF-β1+HSYA)-untransfected group (**Figure 4**). These protein levels were also markedly elevated in the TGF-β1-non-targeting control siRNA compared with the normal-non-targeting control siRNA group, and this elevation was decreased in the (TGF-β1+HSYA)-non-targeting control siRNA group. These data suggest that HSYA can suppress α-SMA, COL1A1, and FN expression induced by TGF-β1. α-SMA, COL1A1, and FN expression were also significantly decreased in the TGF-β1-TβRII siRNA group compared with the TGF-β1-non-targeting control siRNA group, suggesting that these protein levels were decreased by TβRII knockdown. Importantly, there was no significant difference between the TGF-β1-TβRII siRNA and (TGF-β1+HSYA)-TβRII siRNA groups, suggesting that HSYA target TβRII to suppress α-SMA, COL1A1, and FN expression in TGF-β1-treated MRC-5 cells.

### Effects of HSYA on TGF-β1/Smad and ERK/MAPK Signaling Pathway in TGF-β1-Treated MRC-5 Cells with TβRII Knockdown

**FIGURE 5:**
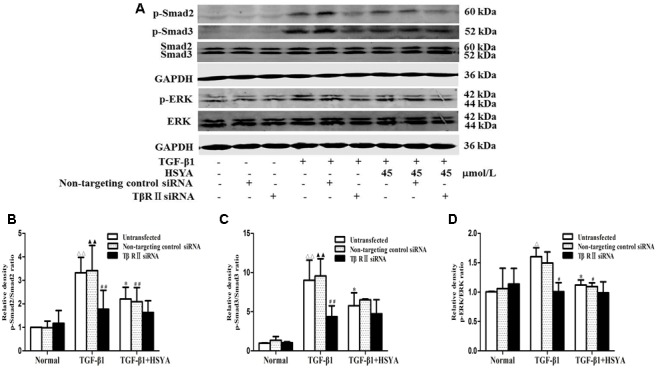
**Effects of HSYA on TGF-β1/Smad and ERK/MAPK signaling pathway in TGF-β1-treated MRC-5 cells with TβRII knockdown.** MRC-5 cells were pretreated with TβRII-non-targeting control siRNA or TβRII siRNA for 24 h followed by 45 μmol/L HSYA for 30 min before stimulation with 10 ng/mL TGF-β1 for 1 h. Protein levels of phosphorylated Smad2, Smad3, and ERK were analyzed by western blotting **(A)**. Densitometric analyses of phosphorylation statuses of Smad2 **(B)**, Smad3 **(C)**, and ERK **(D)**. Data are presented as mean ± SD, *n* = 3 per group. ^ΔΔ^*p* < 0.01 vs. normal-untransfected group, ^▴▴^*p* < 0.01 vs. normal-non-targeting control siRNA,^∗^*p* < 0.05 vs. TGF-β1-untransfected. ^#^*p* < 0.05, ^##^*p* < 0.01 vs. TGF-β1-non-targeting control siRNA group.

We further explored whether HSYA may target TβRII on the cell membrane to attenuate TGF-β1-induced activation of TGF-β1/Smad and ERK/MAPK signaling pathway using TβRII siRNA. Phosphorylation levels of Smad2, Smad3, and ERK were increased in both the TGF-β1-untransfected groups and TGF-β1-non-targeting control siRNA groups (**Figure 5**), and these levels were reduced in the (TGF-β1+HSYA)-untransfected groups and (TGF-β1+HSYA)-non-targeting control siRNA groups, suggesting that HSYA inhibited the phosphorylation of Smad2, Smad3, and ERK induced by TGF-β1. Meanwhile, Smad2, Smad3, and ERK phosphorylation levels were significantly decreased in both the TGF-β1-TβRII siRNA and (TGF-β1+HSYA)-TβRII siRNA groups, with no significant difference between them. This suggested that HSYA targets TβRII to suppress the phosphorylation of Smad2, Smad3, and ERK in TGF-β1-treated MRC-5 cells.

### Effects of HSYA on Binding of FITC-TGF-β1 to MRC-5 Cell Receptors

**FIGURE 6:**
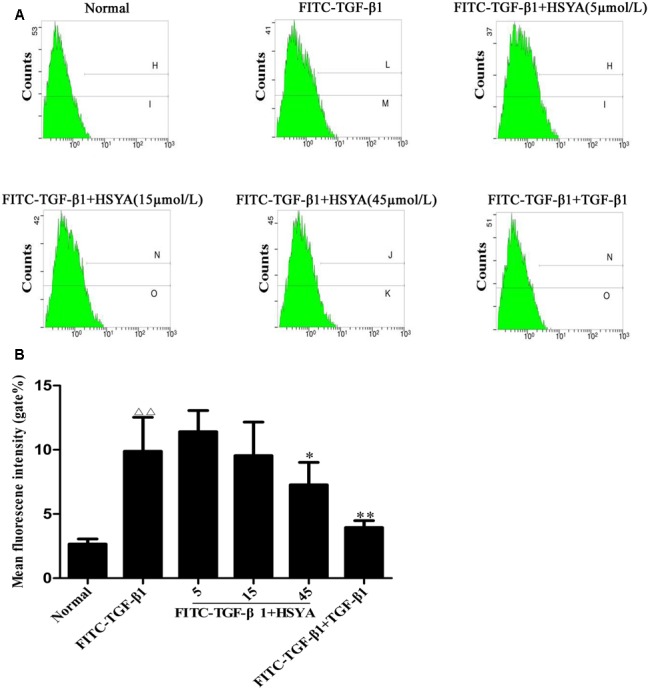
**Antagonistic effects of HSYA on FITC-TGF-β1 binding to MRC-5 cell cytoplasmic receptors.** Binding activity was measured by flow cytometry and determined by cell fluorescence intensity **(A,B)**. The concentration of TGF-β1 was 100-fold that of FITC-TGF-β1. Data presented as mean ± SD, *n* = 6 per group. ^ΔΔ^*p* < 0.01 vs. normal group, ^∗^*p* < 0.05, ^∗∗^*p* < 0.01 vs. FITC-TGF-β1 group.

Fluorescein isothiocyanate-TGF-β1 binding to receptors on MRC-5 cells was measured by flow cytometry. The mean fluorescence intensity (gate %) was increased significantly in the FITC-TGF-β1 compared with the normal group, and this elevation was attenuated in the FITC-TGF-β1+HSYA (45 μmol/L) group (**Figure 6**). The gate % was also attenuated by unlabeled TGF-β1, at a concentration 100 times that of FITC-TGF-β1 in the FITC-TGF-β1+TGF-β1 group. These results suggested that HSYA inhibited FITC-TGF-β1 binding to MRC-5 cell membrane receptors.

## Discussion

Idiopathic pulmonary fibrosis is characterized by progressive ECM deposition, destruction of the lung tissue structure, and ultimate loss of lung function (Verma and Slutsky, 2007; King et al., 2011; Puglisi et al., 2016). During the early stage of lung fibrosis, resident fibroblasts in the lung interstitial space are stimulated and activated (Hoyles et al., 2011), and subsequently proliferate and migrate to the injured regions. Activated fibroblasts also differentiate into myofibroblasts expressing high levels of α-SMA. These cells have the characteristics of mobility and contraction, and can synthesize and secrete large amounts of ECM, leading to the formation of collagen-fiber networks (Tomasek et al., 2002; Hinz et al., 2007; Hinz, 2010). Investigation of methods of interfering with fibroblast activation may thus provide novel therapeutic strategies for the treatment of pulmonary fibrosis.

Transforming growth factor-β1 is considered to be one of the most critical factors responsible for pulmonary fibrosis, with the abilities to promote fibroblast proliferation, migration, and the synthesis of connective tissue components, whilst inhibiting their degradation. TGF-β1 causes lung fibroblasts to be activated and to differentiate into myofibroblasts (Ten et al., 2002; Feng and Derynck, 2005). It has also been shown to mediate the effect of fibrosis through the classical Smad pathway and the non-Smad pathway (Ten et al., 2002; Leask and Abraham, 2004; Guo and Wang, 2009; Biernacka et al., 2011). MRC-5 cells, which retain the features of activated lung fibroblasts including α-SMA expression and the excessive secretion of ECM, are therefore an attractive cell line for use in pulmonary fibrosis research (Ding et al., 2015). In this manuscript, we demonstrated that HSYA inhibited TGF-β1-induced cell proliferation at 48 h or 72 h but not at 24 h. Compared with untreated cells, TGF-β1 stimulation of MRC-5 cells for 24 h did not induce significantly higher OD values in our MTS assay, so an inhibitory effect of HSYA on cell proliferation at this time point could not be assessed. Immunofluorescence staining indicated that HSYA treatment decreased the expression of α-SMA, COL1A1, and FN in MRC-5 fibroblasts. These data were consistent with our previous study (Pan et al., 2016). In addition, the results of the current study showed that HSYA inhibited the effects of TGF-β1 on the migration of MRC-5 embryonic fibroblasts. These results suggest that HSYA may exert an anti-fibrotic role by directly regulating TGF-β1-induced fibroblast proliferation, migration, differentiation, and ECM synthesis. Moreover, these results are in agreement with those of other studies. Yang G.S. et al. (2015) verified that HSYA inhibited the lipopolysaccharide-induced proliferation and migration of vascular smooth muscle cells via a Toll-like receptor-4 pathway. HSYA also suppressed α-SMA and collagen-I expression in a unilateral ureteral obstruction-induced renal fibrosis rat model (Hu et al., 2016), while Jin et al. (2016) demonstrated that HSYA alleviated the BLM-induced increases in TGF-β1, connective tissue growth factor, α-SMA, and collagen I mRNA levels in lung tissue in mice.

The balance between MMPs and TIMPs is important for ECM deposition. MMP-2 and MMP-9, two prominent members of the gelatinase family, are responsible for degradation of collagens types I and IV, respectively. TIMPs inhibit various MMPs; TIMP-1 binds to MMP-9 and TIMP-2 binds to MMP-2 (Björklund and Koivunen, 2005; Bittner et al., 2009). Previous studies reported that HSYA effectively inhibited oxidative stress-mediated hepatic injury partly by up-regulating expression levels of MMP-2, and down-regulating the expression of TGF-β1 and TIMP-1 (Wang et al., 2013). Zhang et al. (2011) found that HSYA reduced collagen A type I, MMP-9, and TIMP-1 gene expression levels in a rat model of hepatic fibrosis induced by carbon tetrachloride, and Wang C.Y. et al. (2014) showed that HSYA decreased gasoline engine exhaust-mediated expression of CD40, MMP-9, ICAM-1, and VCAM-1 in lung tissue. However, we found no effect of HSYA on MMP-2, TIMP-1, and TIMP-2 expression. Further studies are therefore needed to clarify the effects of HSYA on key enzymes in the ECM-degradation process.

Growing evidence has indicated the potential of HSYA as a new therapeutic agent for interfering with the progression of fibrotic disease. For example, Hu et al. (2016) showed that HSYA had a protective effect against fibrosis in renal cells through inhibiting the TGF-β1/Smad3-mediated epithelial–mesenchymal transition signaling pathway, while Zhang et al. (2011) found that HSYA markedly attenuated the development of liver fibrosis by directly blocking TGF-β1-regulated hepatic stellate cell activation. Liu et al. confirmed that peroxisome proliferator-activated receptor-γ and p38 MAPK signaling played pivotal roles in the prevention of liver fibrosis induced by carbon tetrachloride and a high-fat diet (Liu et al., 2014). Jin et al. (2016) recently showed that HSYA attenuated BLM-induced pulmonary fibrosis in mice. A previous pilot study in our lab showed that HSYA inhibition of TGF-β1-induced MRC-5 cell activation was associated with the Smad and ERK/MAPK signaling pathways (Pan et al., 2016). However, there is currently no evidence to explain why HSYA inhibits the TGF-β1 signaling pathway.

To the best of our knowledge, the present study provides the evidence of the mechanisms whereby HSYA inhibits the TGF-β1 signaling pathway. In particular, here we show that HSYA can inhibit both TGF-β1-induced cell proliferation and migration and similar effects were seen with the TβRI kinase inhibitor SB431542, suggesting that HSYA acts on the TβRI signaling pathway.

TβRII is a 567 amino acid single-pass type I membrane protein with a cytoplasmic serine-threonine kinase domain. Upon binding of TGF-β1 to the high-affinity TβRII on the cell membrane, TβRI and TβRII heterodimerize and the serine-threonine kinase TβRI then phosphorylates Smad2/3. Smad2/3 in turn combines with Smad4 and translocates to the nucleus to regulate target genes (Feng and Derynck, 2005). We further elucidated the mechanism whereby HSYA negatively regulated TGF-β1 signaling in MRC-5 cells by focusing on TβRII and TβRI. HSYA had no effect on TGF-β1-induced expression of TβRII or TβRI. Flow cytometry experiments revealed that HSYA could antagonize the binding of FITC-TGF-β1 to MRC-5 cell cytoplasmic receptors. TβRII siRNA comprises a pool of three target-specific 19–25 nucleotide siRNAs designed to knockdown TβRII expression. In this study, we demonstrated that both HSYA and TβRII siRNA inhibited TGF-β1-induced α-SMA, COL1A1, and FN expression, and phosphorylation of Smad2, Smad3, and ERK. Interestingly, a slight decrease was measured in α-SMA and COL1A1 expression in the TGF-β1-non-targeting control siRNA group compared with the TGF-β1 group. However, no significant statistical difference was found in these two groups. The fluctuations of data may have been caused by experimental errors or cell growth state. Furthermore, there was no significant difference in effect between the TGF-β1-TβRII siRNA and (TGF-β1+HSYA)-TβRII siRNA groups, suggesting that TβRII may be the target of HSYA action. HSYA has difficulty penetrating the cell membrane by passive diffusion because of its high water solubility, and it may act on the surface of the cell membrane. Overall, our data indicated that HSYA may exert an inhibitory effect on the TGF-β1 signaling pathway by competitively inhibiting TGF-β1 binding to TβRII, and thus preventing signal transduction by TβRII. TβRII might be the target of HSYA responsible for its inhibition of TGF-β1-induced MRC-5 human fetal lung fibroblast activation.

It has been reported that epithelial–mesenchymal transition (EMT) of lung epithelial cells plays an important role in the process of pulmonary fibrosis (Willis et al., 2005). In our previous work, we demonstrated that HSYA attenuated TGF-β1-induced EMT in A549 cells through suppressing phosphorylation of Smad3 (Jin et al., 2016), but the mechanism and target remain unclear. The sonic hedgehog, Wnt/β-catenin, TGF-β1, epithelial growth factor, fibroblast growth factor, platelet derived growth factor, and vascular endothelial growth factor-mediated signal pathways are all known to be important in cross-talk between the epithelium and the mesenchyme (Chapman, 2011). Future studies directed at understanding how HSYA may modulate those signaling pathways during EMT are critical to fully understanding its biological effects.

In summary, our results suggest that HSYA acts as a therapeutic agent to inhibit the progression of IPF, via suppressing lung fibroblast proliferation, migration, differentiation, and ECM deposition stimulated by the TGF-β1 signaling pathway. We also identified HSYA as an antagonist of the TβRII-mediated activation of MRC-5 cell. Further studies are needed to confirm these results and clarify the precise mechanisms whereby HSYA blocks the TβRII receptor and its downstream signal transduction pathway. HSYA has been used clinically for certain conditions for some time, and now also has the potential to alleviate many kinds of fibrosis.

## Author Contributions

RP carried out the experimental work and wrote the paper. MJ designed and supervised the experiments and revised the primary manuscript. BZ was responsible for analysis of HSYA. YZ and MZ participated in cell culture and molecular biological experiments.

## Conflict of Interest Statement

The authors declare that the research was conducted in the absence of any commercial or financial relationships that could be construed as a potential conflict of interest.

## Supplementary Material

FIGURE S1 **Effects of hydroxysafflor yellow A (HSYA) on MRC-5 cell proliferation.** Cells were pretreated with HSYA (5, 15, and 45 μmol/L) and/or 2 μmol/L SB431542 before stimulation with 1 ng/mL TGF-β1 for 24 h **(A)**, 48 h **(B)**, or 72 h **(C)**. Data are presented as mean ± SD, *n* = 4 per group. ^Δ^*p* < 0.05 vs. normal group, **p* < 0.05, ***p* < 0.01 vs. TGF-β1 group.

FIGURE S2 **Effects of HSYA on myofibroblast activation and ECM deposition in TGF-β1-induced MRC-5 cells.** Cells were treated with 45 μmol/L HSYA or 2 μmol/L SB431542 and then cultured with 10 ng/mL TGF-β1 for 72 h. The expression levels of α-SMA **(A)**, COL1A1 **(B)**, and FN **(C)** were observed by immunofluorescence staining (magnification: 200×, scale bar = 100 μm). *n* = 4 per group.
