# Supplementary figures and images for: Hydroxysafflor Yellow A Suppresses MRC-5 Cell Activation Induced by TGF-β1 by Blocking TGF-β1 Binding to TβRII
Source: Front Pharmacol. 2017 May 11;8:264. doi: 10.3389/fphar.2017.00264 (PMC5425600; doi:10.3389/fphar.2017.00264)

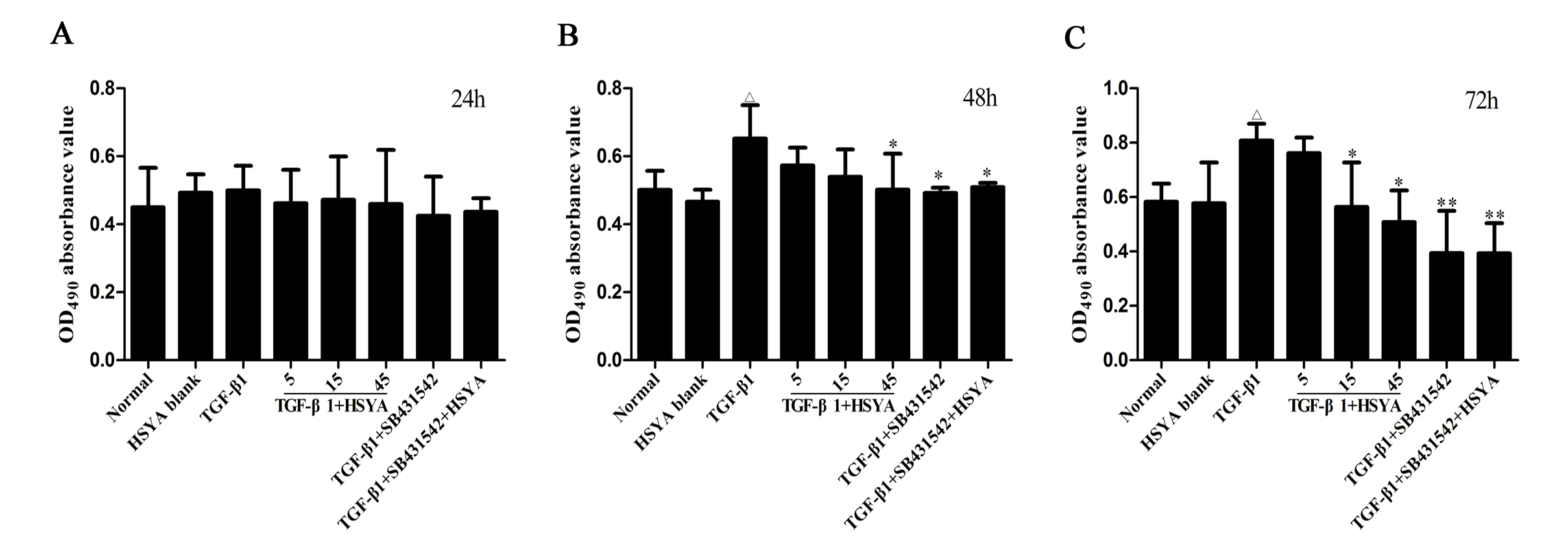

Supplement: FIGURE S1 — Effects of hydroxysafflor yellow A (HSYA) on MRC-5 cell proliferation. Cells were pretreated with HSYA (5, 15, and 45 μmol/L) and/or 2 μmol/L SB431542 before stimulation with 1 ng/mL TGF-β1 for 24 h (A), 48 h (B), or 72 h (C). Data are presented as mean ± SD, n = 4 per group. Δp < 0.05 vs. normal group, *p < 0.05, **p < 0.01 vs. TGF-β1 group. [file Image_1.TIF]

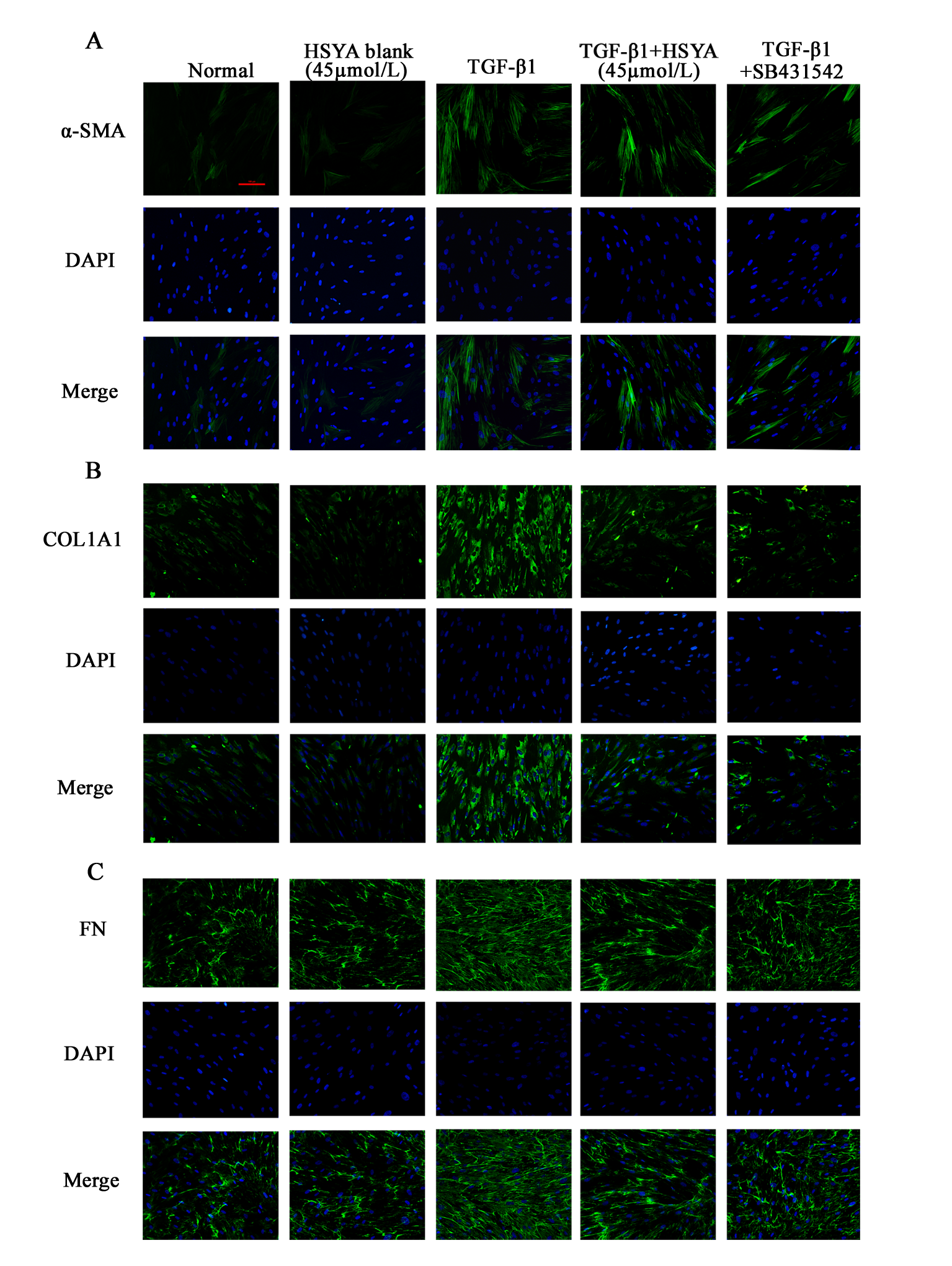

Supplement: FIGURE S2 — Effects of HSYA on myofibroblast activation and ECM deposition in TGF-β1-induced MRC-5 cells. Cells were treated with 45 μmol/L HSYA or 2 μmol/L SB431542 and then cultured with 10 ng/mL TGF-β1 for 72 h. The expression levels of α-SMA (A), COL1A1 (B), and FN (C) were observed by immunofluorescence staining (magnification: 200×, scale bar = 100 μm). n = 4 per group. [file Image_2.TIF]
